# Induction of anterior gradient 2 (AGR2) plays a key role in insulin-like growth factor-1 (IGF-1)-induced breast cancer cell proliferation and migration

**DOI:** 10.1007/s12032-015-0577-z

**Published:** 2015-05-09

**Authors:** Zheqi Li, Zhenghua Wu, Hao Chen, Qi Zhu, Guangwei Gao, Lingyun Hu, Hema Negi, Suchitra Kamle, Dawei Li

**Affiliations:** School of Pharmacy, Shanghai Jiao Tong University, 308-Building#6, 800, Dongchuan Rd., Shanghai, 200240 China

**Keywords:** Anterior gradient 2, Insulin-like growth factor-1, Breast cancer, Estrogen response element, Activator protein-1 site

## Abstract

Anterior gradient 2 (AGR2) is a promising anti-tumor target associated with estrogen receptor expression and metastatic progression of breast cancer. Insulin-like growth factor-1 (IGF-1) is another potent factor that stimulates breast cancer progression and mediates anti-estrogen drug resistance. However, the precise mechanism and connections between these two factors in breast cancer drug resistance have not been fully elucidated. Here, for the first time, we decipher that IGF-1 remarkably induces AGR2 in the MCF7 cell line, through an estrogen response element (ERE) between −802 and −808 bp and a leucine zipper transcription factor-binding site located between −972 and −982 bp on the AGR2 promoter. We also found that the ERK1/2 and AKT pathways mediate estrogen receptor-α at the upstream of ERE and that the JNK pathway activates the leucine zipper site through the c-Jun/c-Fos complex. Additionally, our data suggest that knockdown of AGR2 reduces IGF-1-induced cell proliferation, migration and cell cycle progression. Therefore, we report that AGR2 is a key modulator involved in IGF-1-induced breast cancer development. We propose that the identification of the mechanism linking the IGF-1/insulin signal and AGR2 promoter activation is important, because it provides insights into the development of anti-breast cancer drugs.

## Introduction

Anterior gradient-2 (AGR2), also known as HAG-2, is the human homologue of *Xenopus laevis* cement gland protein XAG-2 [1] and nAG, which is responsible for limb regeneration in the newt [2]. AGR2 has been reported to be overexpressed in several human cancers [3], including estrogen receptor (ER)-positive breast cancer. AGR2 overexpression in breast cancer cells promotes cell migration and malignant transformation [4]. The clinical and prognostic significance of AGR2 has been demonstrated, suggesting its potential as a tumor diagnostic marker [5] and a modulator in breast cancer anti-estrogen drug resistance [6].

However, the full spectrum of factors and mechanisms regulating AGR2 levels in tumor cells are still poorly understood. Strikingly, AGR2 expression is increased over sevenfold in the presence of estrogen in hormone-dependent breast cancer cells [7]. A possible mechanism was recently proposed to explain this estrogen-induced expression of AGR2 [8]. In that study, AGR2 expression was reported to be activated by estradiol, through the binding of ER to the estrogen response elements (ERE) located in the potential transcriptional regulatory region of AGR2 promoter. In addition to the estrogen receptor, current research on AGR2 using promoter analysis also identified the transcription factors Foxa1 and Foxa2 as major players in regulating AGR2 promoter activity [9]. The ErbB3-binding protein 1 (EBP1)—Foxa signal circuit [10]—was also reported as playing a significant role in AGR2 promoter modulation.

The insulin-like growth factor (IGF-1)/insulin signaling axis has widely been reported in modulating breast cancer anti-estrogen drug resistance [11]. Binding of IGF-1 to its receptor activates receptor tyrosine kinase activity, leading to the phosphorylation of key downstream effectors such as insulin receptor substrate proteins-1 (IRS-1) [12] and insulin receptor substrate proteins-2 (IRS-2) [13] as well as Src homology collagen (SHC) [14]. Significantly, cross talk between the IGF-1 receptor and estrogen receptor-α has also been reported as a requirement for the rapid activation of the IGF-1 receptor via specific signaling cascades in breast cancer [15]. In addition, many pathways have been reported to be highly involved in IGF-1-induced anti-estrogen drug resistance, including PI3K/AKT [16] and mTOR [17]. However, the specific modulator related to IGF-1-induced breast cancer development is still poorly understood.

Our findings suggest AGR2 induction as a novel IGF-1-induced breast cancer formation mechanism, relevant to both the activation of the ER and the non-ER pathways. These results provide further insights toward the development of anti-estrogen drug-resistant tumors and their potential therapeutic targets.

## Materials and methods

### Cell culture and treatment

The MCF7 cell line (ATCC HTB-22) was obtained from the American Type Culture Collection (ATCC, Manassas, VA, USA). Cells were cultured in Dulbecco’s modified Eagle’s medium (DMEM, Invitrogen) with 10 % fetal bovine serum (FBS, Gibco Life Technologies) and penicillin streptomycin (Solarbio). Before treatment, cells were transferred from 10-cm plates to 6-well plates and were serum-starved for 24 h with DMEM without phenol red (Gibco Life Technologies). Treatments included β-estradiol (E2, Sigma-Aldrich), insulin-like growth factor-1 (IGF-1, Genescript) and insulin (Solarbio) in the same medium for 24 h or 15 min. Anti-estrogen drugs include tamoxifen and doxorubicin from Thermo, as well as raloxifene and fulvestrant from Selleckchem. In addition, chemical inhibitors included U0126-EtOH (MEK inhibitor), fulvestrant (ICI 182,780, estrogen receptor-α inhibitor) and MK2206 2HCl (AKT inhibitor), and SP00125 (JNK Inhibitor) and OSI-906 (IGF-1 receptor inhibitor), which were also purchased from Selleckchem, while monensin was from Beyotime.

### Protein extraction and western blotting

After treatment, cells were lysed in NP-40 lysis buffer. Proteins were separated using SDS-PAGE and transferred onto nitrocellulose membrane for antibody detection. The primary antibodies included β-actin from Santa Cruz Biotechnology; AKT and phosphor-AKT from Epitomics; and p44/42 MAPK, phosphor-p44/42 MAPK, estrogen receptor-α, phosphor-estrogen receptor-α and JNK from Cell Signal Technology. In addition, the monoclonal antibody against AGR2, 18A4, was prepared by our laboratory. The secondary antibodies included goat anti mouse and rabbit from Odyssey. Fluorescence was detected with infrared imaging scanner (Odyssey).

### shRNA knockdown

AGR2 knockdown in MCF7 cells was performed using an isopropyl β-D-1-thiogalactopyranoside-inducible AGR2 shRNA lentiviral construct. Cells were infected with AGR2 shRNA lentiviral construct or a control vector and selected using puromycin after 48 h. Stably transfected cell lines were grown in the presence of 1 mM β-D-1-thiogalactopyranoside for 7 days to induce shRNA expression.

### iCell proliferation assay

Baselines were first measured by adding DMEM into iCelligence (ACEC Biosciences) sensor wells. Cells were counted first with a hemocytometer and added to each sensor well up to 10,000 cells per well. After the curves showed stable changes, which indicate cell adherence, different treatments were added to each well. The proliferation rates are indicated by the slope of the growth curve.

### Flow cytometry assay

Cells were treated in different conditions and fixed in 70 % ethanol for 12 h. The samples were analyzed using a FACSCanto flow cytometer (Becton–Dickinson), and FlowJo software was used to analyze the cell cycle distribution.

### Plasmid construction and mutagenesis preparation

The AGR2 promoter-luciferase (AL) plasmid was constructed by ligation of pGL3-basic vector and AGR2 sequence, and mutagenesis was performed using the KOD-Plus-Mutagenesis Kit (Toyobo). The sequences of the designed mutagenesis primers are shown in Table 1. The pGL3-basic vector (Promega) without the AGR2 promoter sequence served as a negative control.

**Table 1:** Sequence of primers of different mutagenesis on AGR2 promoter

Mutagenesis	Primer sequence
D1	Antisense: 5′ CTAGCACGCGTAAGAGCTCGGTACC 3′ Sense: 5′ GTCATTTAATATTCAAAATGGTCCC 3′
D2	Antisense: 5′ CTAGCACGCGTAAGAGCTCGGTACC 3′ Sense: 5′ GAATTGAAAGGAAATTCAGTATT 3′
D3	Antisense: 5′ CTAGCACGCGTAAGAGCTCGGTACC 3′ Sense: 5′ CTCAGTTTTGAAAAATTACGTGGG 3′
FL-Δ1	Antisense: 5′ GACTTTTCCCTGTATTGCCACATGG 3′ Sense: 5′ CTATGAGCAAGCACCAGGATGCAGG 3′
FL-Δ2	Antisense: 5′ GATCTGAGATCTCATTTAACATCCC 3′ Sense: 5′ GTCCCAACTCTGCCCTAAACTAGTT 3′
FL-Δ3	Antisense: 5′ CAAAGTCCTTCAGTCCCTTATCCAC 3′ Sense: 5′ TATTACAAGGGTCTATCTAAGGGCC 3′
FL-Δ4	Antisense: 5′ CAATTCCAGTCTTTCATTTTACAGATG 3′ Sense: 5′ GAAAGGAAATTCAGTATTTGGAGAATC 3′
FL-Δ5	Antisense: 5′ CTAATGAATTTATAGAAGTAATTTCTTC 3′ Sense: 5′ CATTTTAAAAAGTCATTTATATAGG 3′
FL-Δ6	Antisense: 5′ GAGTTAAGGTCATAATATTTCAAAAAC 3′ Sense: 5′ GCACACAACTTCATGAACAAAATAC 3′
FL-Δ7	Antisense: 5′ GCAGTCTTTAAAAGCTCAGAATGAAG 3′ Sense: 5′ GGGAAAAAAAACTTGGTTGCAGACC 3′
FL-Δ8	Antisense: 5′ CTCAAGACCATTTAATTACTCCCTG 3′ Sense: 5′ CTGTGAAATACCTTTGAACTCTGTG 3′
FL-Δ9	Antisense: 5′ CTTATTTAAAGGCAAACTTTCCTGC 3′ Sense: 5′ GACAGGAGCAGGGAAGTATTGTAG 3′
M1	Antisense: 5′ CGGCAATTCCAGTCTTTCATTTTAC 3′ Sense: 5′ CCGACAAGGGTCTATCTAAGGGCCTG 3′
M2	Antisense: 5′ CGCCAATTCTTAAAACTAGCAGGCC 3′ Sense: 5′ GGCCATCATCGTTTTCAAACTCCTG 3′
M3	Antisense: 5′ GCGATTTGAGCAAAATTCTTAAAACTAG 3′ Sense: 5′ GCGTTTCAAACTCCTGAAGAAATTACTTC 3′
M4	Antisense: 5′ GGGCTTCAGGAGTTTGAAAACGATG 3′ Sense: 5′ GGGCTTCTATAAATTCATTAGAATTGAAAGG 3′
M5	Antisense: 5′ GGGGTATAGAAGTAATTTCTTCAGGAG 3′ Sense: 5′ GGGGAGAATTGAAAGGAAATTCAGT 3′
M6	Antisense: 5′ CGAACTTGAATTCTGTGGGCAAAATC 3′ Sense: 5′ GGGTGGAAAAATATACATACTTGCAAATG 3′
M7	Antisense: 5′ GGAATAAATCCTTGAATTCTGTGGG 3′ Sense: 5′ GGAAATATACATACTTGCAAATGTTTTTG 3′
M8	Antisense: 5′ GGGGAAAAACATTTGCAAGTATG 3′ Sense: 5′ GGGTATGACCTTAACTCATTTTAAAAAG 3′
M9	Antisense: 5′ GGGTAATATTTCAAAAACATTTGCAAG 3′ Sense: 5′ GGGGAACTCATTTTAAAAAGTCATTTATATAG 3′

The primers sequences in the above table are used for PCR process of preparation of corresponding mutagenesis

### Transfection and dual-luciferase assay

The transfection was performed using PEI (Polysciences, Inc.) according to the CELLTECH’s protocol with modification. A reporter plasmid (2 μg) with 0.2 μg of pRL-TK (Promega) served as an internal control for each well in 6-well plates. In 5 h after transfection, the cells were incubated in fresh DMEM for over 12 h and transferred to 96-well plates. Then, the cells were exposed to different treatments for 24 h and assayed for luciferase activity using a dual-luciferase assay system (Promega). The luciferase intensity of the reporter gene and pRI-TK were measured with an automatic-sampling microplate reader (Thermo Fisher Scientific).

### RT-PCR

Total RNA was isolated from cells after 24 h of treatment, and the RNA content was measured. Reserve transcription was performed followed by ReverseTra Ace qPRC RT Kit (Toyobo). The gene-specific RT-PCR, targeting AGR2 and GAPDH, was conducted with ThunderBird SYBR qPCR Kit (Toyobo) and Applied Biosystems Real-Time PCR Instrument (Life Technology). The sequences of the designed mutagenesis primers are shown in Table 2. The mRNA fold changes were calculated according to the ΔΔ*Ct* value.

**Table 2:** Primers sequence of RT-PCR assay

RT-PCR target	Primer sequence
AGR2	Antisense: 5′ GGAGGACAAACTGCTCTGCCAA 3′ Sense: 5′ TCCAAGACAACAAACCCTTG 3′
BCAR1	Antisense: 5′ CTGGAAAAGGGCAGCATCAC 3′ Sense: 5′ CTATGGGCCGTGACACCTC 3′
BCAR2	Antisense: 5′ CTATGGTCATGCCCGTGTCTG 3′ Sense: 5′ TTCCTGGGGATTTCGAGCAC 3′
GAPDH	Antisense: 5′ TGATGGCATGGACTGTGGTCATGAG 3′ Sense: 5′ CTCCTGCACCACCAACTGCTTAGC 3′

The primers sequences in the above table are used for RT-PCR process for detecting the mRNA level of target gene

### Immunofluorescence assay

MCF7 cells grown on coverslips were fixed with 4 % formaldehyde for 10 min and blocked with goat serum for 30 min. Coverslips were incubated with the AGR2 antibody for 2 h and washed with PBS. They were incubated with secondary antibody conjugated with Dylight488 (MutiSciences Biotech). Nuclei were counterstained using DAPI (Invitrogen). The fluorescence was observed and captured with laser confocal microscopy (Leica Camera).

### Wound-healing assay

MCF7 cells were seeded on a 6-well plate with a near 100 % intensity and starved in serum-free DMEM for 12 h. Gaps were created in a cross shape with pipette tips in the middle of each well. Images of each gap were captured 0 and 24 h after different treatments. The migration rate was calculated by the ratio of average migration distance at 24 h to the original gap distance at 0 h, measured in pixels.

### Statistical analysis

All values are expressed as the mean ± standard deviation (SD). The Origin 9.0 Pro program (OriginLab, US) was used for all statistical analyses and graph drawing. Two-tailed Student’s *t* test was used to compare measurements of pairs of samples if appropriate.

## Results

### IGF-1 induces AGR2 expression in a dose-dependent manner in MCF7 cells

**Fig. 1:**
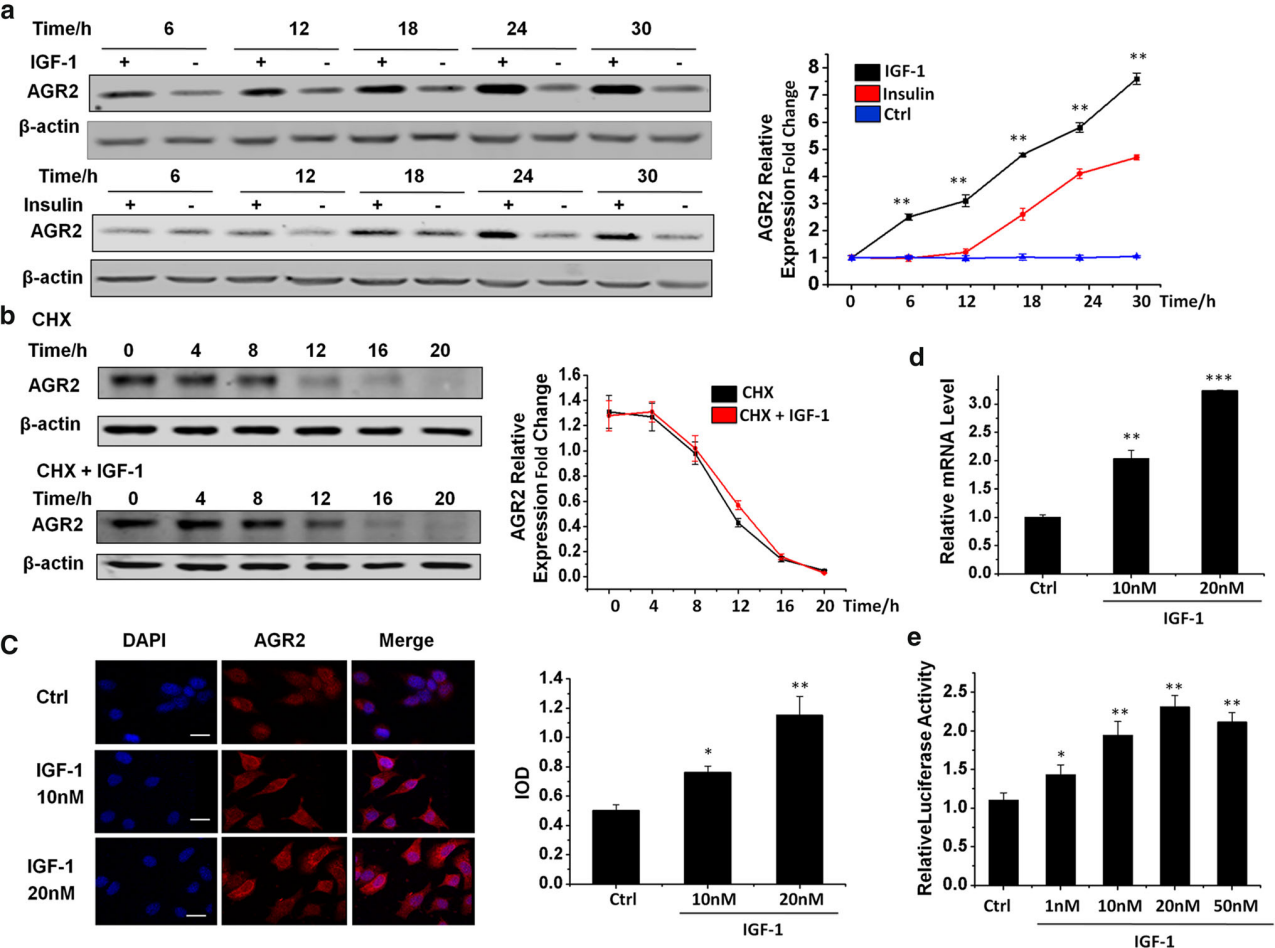
IGF-1 induces AGR2 expression in a dose-dependent manner in MCF7 cells. **a** MCF7 cells were treated with 10 nM IGF-1 and 5 μg/ml insulin for five different time intervals (6, 12, 18, 24 and 30 h). AGR2 levels were determined with a western blot assay. **b** AGR2 expression induced by IGF-1 without E2 was detected via immunofluorescence in MCF7 cells using confocal microscopy. Cells were starved for 24 h before IGF-1 treatment. The nuclei were stained with DAPI (*blue*) as an internal reference, and AGR2 was stained with specific primary antibody (*red*). The IOD value of *red* fluorescence was quantified using Image-Pro, normalized to *blue* fluorescence and set in the *right panel* in the form of a histogram. The original magnification is ×400. **c** MCF7 cells were treated with 20 μg/ml CHX with or without 10 nM IGF-1 for six different time periods (0, 4, 8, 12, 16 and 20 h). A western blot assay was utilized to show the degradation rate of AGR2 in both groups. Relative AGR2 levels are shown in the *right panel*. **d** Relative AGR2 mRNA level of MCF7 cells compared with GAPDH was determined using RT-PCR after 24 h of treatment with 10 and 20 nM IGF-1. **e** Dose-responsive IGF-1 activation of AGR2 transcription was confirmed by a 1.9 kb of AGR2 promoter-luciferase reporter assay with a pRL-TK plasmid co-transfected into MCF7 cells and treated with four different concentration of IGF-1 (1, 10, 20 and 50 nM). The luciferase intensity was detected with a microplate reader and normalized to the intensity of Renilla. The pGL3-basic vector was detected as a negative control. Each experiment was repeated at least three times. *P < 0.05; **P < 0.01; ***P < 0.0001

It has been widely reported that AGR2 can be induced in the exposure of hormones [18] or physiological stress [19]. Here, we treated MCF7 cells with a significant breast cancer-related growth factor, IGF-1, in a time course, from 6 to 30 h. We compared the induction level with insulin, a structural analogue of IGF-1 (Fig. 1a). The data indicated that IGF-1 has a more rapid and stronger AGR2 induction than insulin in MCF7 cells. In addition, an immunofluorescence assay revealed that the IGF-1 induction of AGR2 occurred in a dose-dependent manner at protein level (Fig. 1b).

To determine whether this increase in AGR2 expression caused by IGF-1 is relevant to protein degradation, we performed a cycloheximide degradation assay (Fig. 1c). The relative AGR2 level descent did not show any significant difference in the presence of IGF-1. We also detected this induction using an RT-PCR assay (Fig. 1d). The results confirmed the IGF-1 dose-dependent induction of AGR2 at the mRNA level. To further determine whether the dose-dependent induction also occurred at the transcriptional level, we used a 1.9 kb AGR2 promoter-luciferase construct to study the activation effect of IGF-1 (Fig. 1e). The promoter activity also increased in the presence of IGF-1 in a dose-dependent manner, with maximum stimulation at 20 nM. To conclude, IGF-1 shows a significant induction of AGR2 at the expression, the transcription and the mRNA level rather than due to changes in degradation.

### An estrogen response element and a leucine zipper-binding site both are required for AGR2 promoter activation by IGF-1

**Fig. 2:**
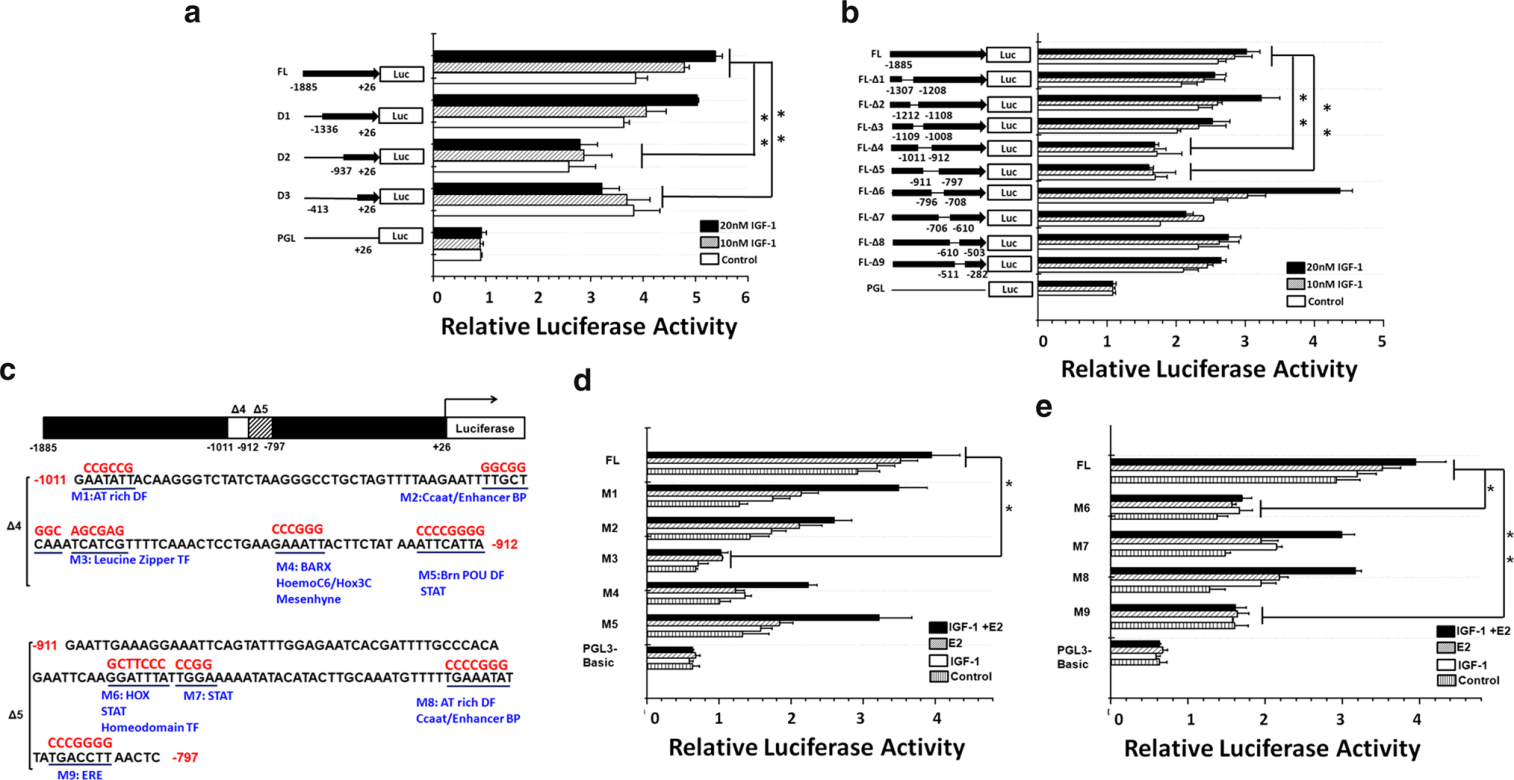
An estrogen response element and a leucine zipper-binding site are both required for AGR2 promoter activation by IGF-1. **a** AGR2 promoter deletion mutations were analyzed by luciferase reporter assays. Luciferase reporter constructs containing different length of AGR2 promoter sequence were co-transfected with pRL-TK control plasmid into MCF7 cells. Cells were then treated with 10 nM IGF-1 and 20 nM IGF-1 for 18 h without E2. The fluorescence intensity of cell extracts was recorded by microplate reader and normalized by control Renilla luciferase activity. **b** AGR2 promoter with 100-base pair scanning deletions were analyzed for IGF-1 effects similarly as in (**a**). **c** The Transfac database was used to analyze the potential transcriptional binding sites indicated within the target sequences identified by (**a**, **b**), and site-specific mutagenesis were designed based on the result of bioinformatics analysis. **d**, **e** Sequence-specific mutations designed to destroy potential transcription factor-binding sites in D4 (**d**) and D5 (**e**) according to (**c**) were used in this promoter assay. After transfection, the cells were treated with control PBS, 10 nM IGF-1, 5 nM E2 and the mixture of IGF-1 and E2 for 18 h. The pGL3-basic vector was used as a negative control. Each experiment was repeated at least three times. *P < 0.05; **P < 0.01; ***P < 0.0001. TF

There are abundant reports on the modulation of AGR2 expression in breast cancer [20]; however, AGR2 promoter activation by IGF-1 has not been reported. Therefore, we further investigated the specific modulation sites on the AGR2 promoter responsive to IGF-1 induction. A series of AGR2 promoter deletion mutations linked to luciferase reporter were constructed to identify potential IGF-1-responsive elements (Fig. 2).

Deletion from −1336 to −413 abolished the IGF-1 effect on AGR2 promoter (Fig. 2a), indicating the existence of a major responsive element in this segment. We further identified two different regions that may be responsible for IGF-1 activation, −1011 to −912 (Δ4) and −911 to −797 (Δ5). A scanning deletion mutagenesis with approximately 100-base pair overlapping deletions from −1307 to −282 was performed on the AGR2 promoter. (Figure 2b).

Further identification of the potential roles of transcription factor-binding sequences was conducted by site-specific mutagenesis, which abolished 9 transcription factor-binding sites in the Δ4 and Δ5 regions. We identified them through the analysis of the transcription factor-binding site’s database Transfac [21] (Fig. 2c). Analysis of these promoter mutations showed that three transcriptional regulatory elements were critical, to different degrees, for IGF-1 and E2 activation of the AGR2 promoter in the Δ4 region (Fig. 2d) and in the Δ5 region (Fig. 2e).

According to the results shown in Fig. 2d, mutation M3, which abolished a leucine zipper element, also abolished the IGF-1 stimulation effect. The leucine zipper element is suggested to be critical for the IGF-1-induced response. Figure 4E showed that M6, which lost the HOX-PBX/homeodomain/STAT complex-binding site between −894 and −888 bp, also showed a reduction approximately 50 % in promoter activity in response to both IGF-1 and E2. With the abolishing of a highly homologous estrogen response element located between −808 and −802 bp, M9 showed almost no response to either E2 or IGF-1 stimulation. The result suggested that this ER-binding site is indispensible in E2- and IGF-1-induced AGR2 transcriptional regulation, similar to M6.

### Estrogen-independent activation of AGR2 by IGF-1 requires estrogen receptor and activation of ERK and AKT pathways

**Fig. 3:**
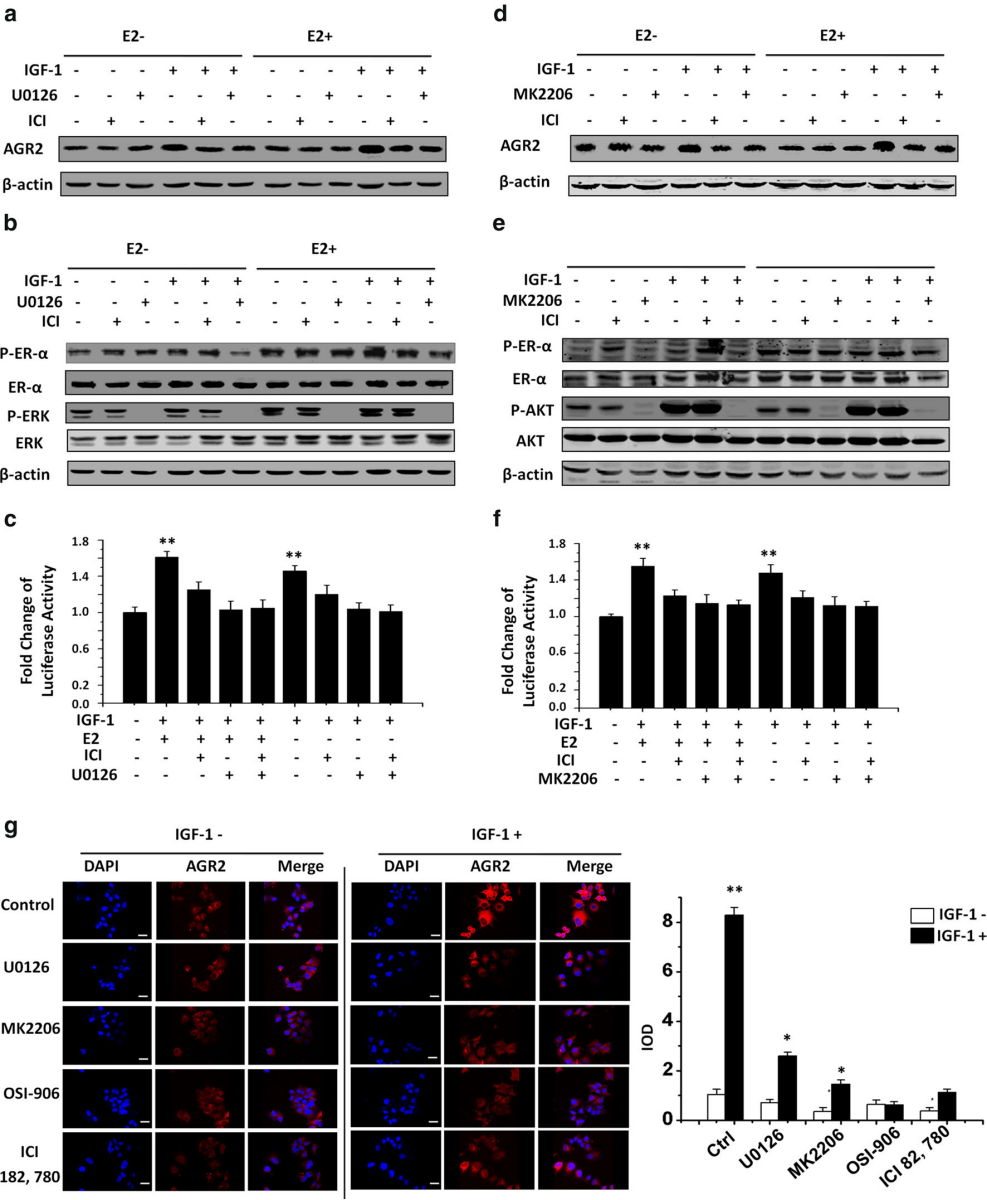
Estrogen-independent activation of AGR2 by IGF-1 requires estrogen receptor and activation of ERK and AKT pathways. **a**, **b** and **d**, **e** MCF7 cells were incubated for 24 h in different treatments with IGF-1 and MEK, ER and AKT inhibitors. The concentration of IGF-1 is 10 nM in each group. MEK inhibitor U0126 (10 μM), ER inhibitor ICI 182, 780 (10 nM) and AKT inhibitor MK2206 (500 nM) and E2 (5 nM) were used. Level of AGR2 protein was detected by western blot with specific antibodies. β-Actin was detected as a loading control. **c**, **f** Luciferase construct with 1.9 kb of AGR2 promoter sequence and a pRL-TK control plasmid was co-transfected into MCF7 cells and treated with different inhibitors with or without the presence of 10 nM IGF-1 and 5 μM E2. The luciferase activity was detected with microplate reader and normalized by Renilla activity. **g** AGR2 expression induced by IGF-1 without E2 in the presence of four chemical inhibitors was detected by immunofluorescence in MCF7 cell by confocal microscopy. The inhibitor concentration was 10 μM U0126, 10 nM ICI 182,780, 500 nM MK2206 and 5 μM OSI-906. Cells were starved for 24 h before IGF-1 treatment. Nuclei were stained with DAPI (*blue*) as an internal reference, and AGR2 was stained with specific primary antibody (*red*). The IOD value of red fluorescence is quantified by Image-Pro, normalized to blue fluorescence and set in the *right panel* in the form of a histogram. The original magnification is ×200. Each experiment was repeated at least three times. *P < 0.05; **P < 0.01

To further elucidate AGR2 activation through the IGF-1 signaling cascade upstream of estrogen response element on the AGR2 promoter, inhibitors for the key components of the ERK, AKT and estrogen pathways were used in a series of western blot and luciferase assays (Fig. 3). The results revealed that IGF-1 induces AGR2 expression with or without E2. Additionally, the inhibition of MEK, a key upstream regulator of ERK1/2, by U0126 or the inhibition of ER by ICI 182, 780, an ER inhibitor also called Fulvestrant, partially abrogated AGR2 induction by IGF-1 in western blot analysis (Fig. 3a).

We also examined the status of ER and ERK with IGF-1 and inhibitor treatments. Although U0126 treatment reduced ER phosphorylation, ER inhibition by ICI did not significantly reduce phosphorylated ERK, indicating that ER phosphorylation is downstream of ERK phosphorylation (Fig. 3b). An AGR2 promoter-reporter assay further confirmed the effects, showing that inhibition at the transcriptional level (Fig. 3c).

Another significant pathway we investigated was the AKT pathway, which has been well reported as a key substrate downstream of the IGF-1 receptor [22]. We used the AKT inhibitor, MK2206, to identify the role of this pathway in IGF-1/AGR2 signaling. A western blot assay for AGR2 indicated that either the AKT or ER pathway abolished the ability of IGF-1 to up-regulate AGR2 expression. The results pointed out that AKT pathway also participates in IGF-1–AGR2 regulation (Fig. 3d). A western blot for phosphorylated ER and AKT revealed that inhibition of AKT abolished ER phosphorylation, whereas inhibition of ER did not influence the AKT phosphorylation. It suggested that ER activation is downstream of AKT phosphorylation in IGF-1 signaling (Fig. 3e). To confirm this result at the transcriptional level, a luciferase assay was conducted. The result showed a striking abolishment of IGF-1 stimulation under the treatment of ICI, MK2206 or both at the transcriptional level (Fig. 3f).

To further investigate the contribution of these signaling pathways in AGR2 induction by IGF-1, we used an in situ immunofluorescence assay with four chemical inhibitors to detect the AGR2 expression (Fig. 3g). The results showed that the blockage of ERK, AKT and ER-α can partially inhibit the IGF-1 induction of AGR2, whereas blocking IGF-1 receptor fully inhibited this effect. In conclusion, the data indicated that AGR2 induction by IGF-1 was entirely through the IGF-1 receptor pathway, which involved ERK, AKT and ER pathways.

### IGF-1 stimulates AGR2 expression through AP-1 site on the AGR2 promoter

**Fig. 4:**
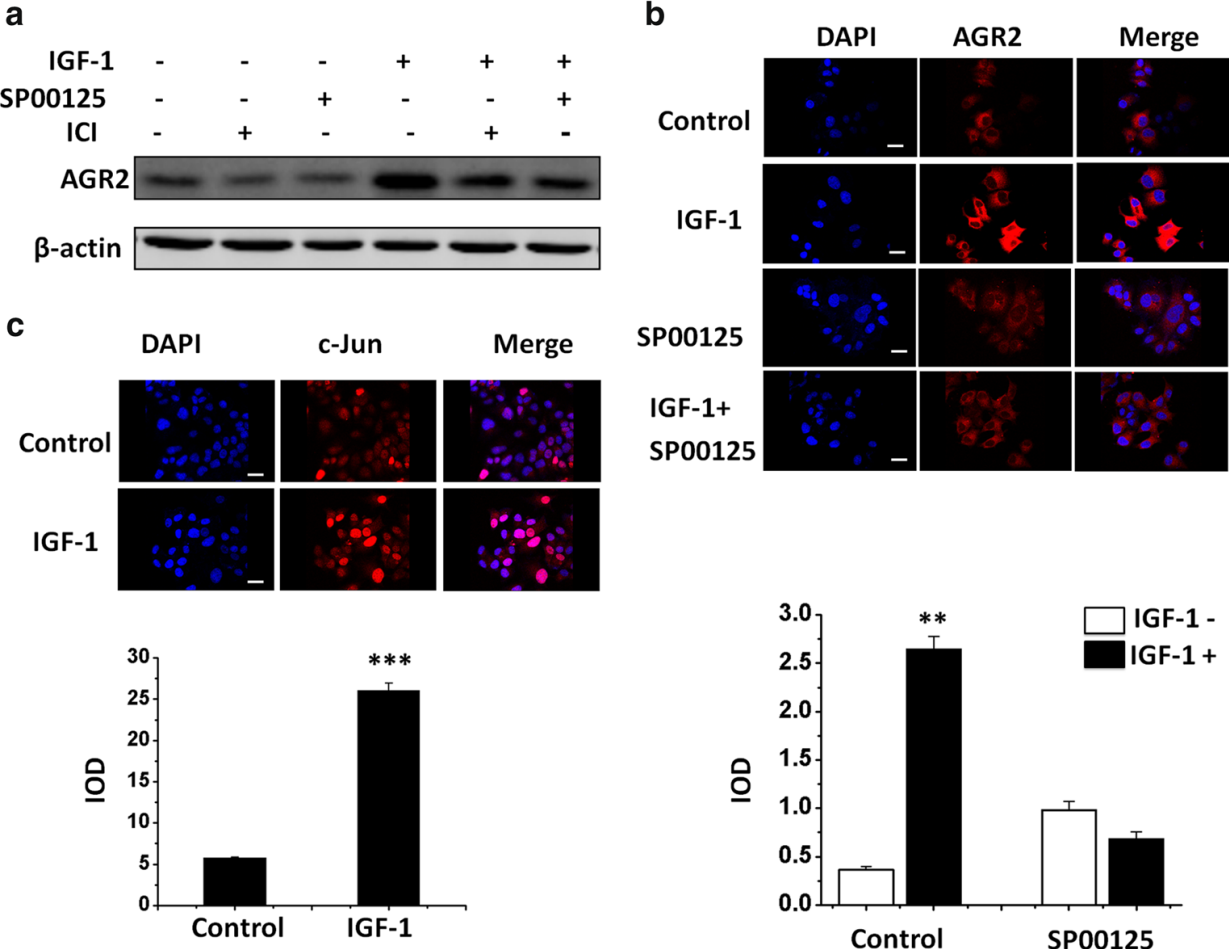
IGF-1 stimulates AGR2 expression through AP-1 site on the AGR2 promoter. **a** MCF7 cells were incubated for 24 h in different treatments with IGF-1 and JNK and ER inhibitors. The concentration of IGF-1 is 10 nM in each group. SP00125 at the concentration of 5 μM was used. Level of AGR2 protein was detected by western blot with specific antibodies. β-Actin was detected as a loading control. **b**, **c** AGR2 expression induced by IGF-1 without E2 in the presence of four chemical inhibitors was detected by immunofluorescence in MCF7 cell by confocal microscopy. The inhibitor, SP00125, concentration was 5 μM. Cells were starved for 24 h before IGF-1 treatment. Nuclei were stained with DAPI (*blue*) as an internal reference, and AGR2 and c-Jun were stained with specific antibody (*red*). The IOD value of *red* fluorescence is quantified by Image-Pro, normalized to *blue* fluorescence and set in the *right panel* in the form of a histogram. The original magnification is ×200 for AGR2 and ×100 for c-Jun. Each experiment was repeated at least three times. *P < 0.05; **P < 0.01

In addition to the estrogen response element, we also identified the leucine zipper transcription-binding site as a potential transcriptional element. Among all potential leucine zipper-binding sites, the AP-1-binding site associated with c-Jun/c-Fos through the JNK pathway [23] has been widely reported to relate to IGF-1 receptor [24]. Therefore, we used Sp00125, a selective JNK inhibitor, to investigate the pathway with western blot and immunofluorescence assays (Fig. 4).

The result reveals that the inhibition of both c-Jun at the downstream and ER-α with Sp00125 or ICI 182, 780 partially abrogate the induction of AGR2 by IGF-1(Fig. 4a). This abrogation effect on AGR2 induction by c-Jun inhibition was confirmed by an in situ immunofluorescence assay in MCF7 cells (Fig. 4b). Furthermore, in an immunofluorescence assay on the location changes of c-Jun, we showed that IGF-1 remarkably enhanced c-Jun translocations into the nucleus (Fig. 4c). Therefore, we propose that the leucine zipper transcription-binding site is actually an AP-1 site, and it can be specifically activated through the JNK pathway downstream of the IGF-1 receptor.

### AGR2-knockdown reduces IGF-1-induced cell proliferation, migration and cell cycle progression

**Fig. 5:**
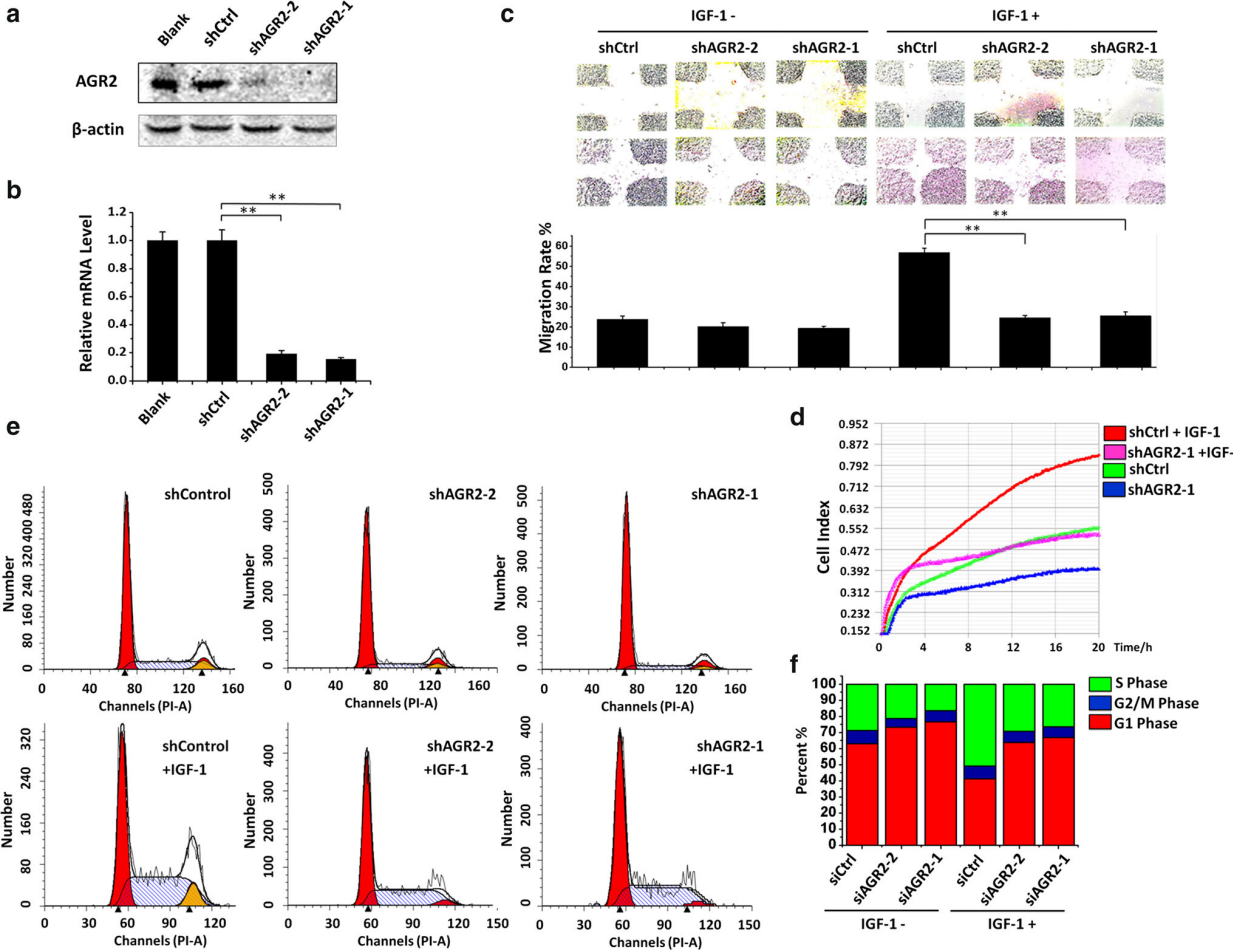
AGR2 is required for IGF-1-induced cell proliferation, migration and cell cycle progression. **a** MCF7 cells were transfected with AGR2 shRNAs or a control shRNA and grown for 14 days in puromycin selection. Western blotting of day 14 whole-cell lysates was performed using AGR2 monoclonal antibody that recognizes AGR2. β-Actin was used as a loading control. **b** RNA was isolated from two clones of shAGR2 cells and shControl cells. cDNA was prepared with a reverse PCR kit and used for real-time PCR with SYBR dye and specific primers. The ΔΔCt values in shAGR2 cells were calculated to show the fold increase compared with shControl cells. GAPDH was used as an internal control. **c** shAGR2 and shControl cells were seeded into six-well plates. A wound in the cells was created with a pipette tip after adherence. The wound widths were recorded under microscopy at 0 and 24 h after treatment with or without 10 nM IGF-1. The migratory distances of all four fringes were then measured according to the pixels on the image directly, and the migration rates were calculated according to the average ratio of migration distances to original wound widths. **d** An iCell proliferation assay was performed on shAGR2 and shControl cells with or without 10 nM IGF-1 treatment. Cell index proliferation curves were generated from 20 h of real-time monitoring. **e** shAGR2 and shControl cells were seeded into six-well plates and starved in serum-free medium for 24 h. Then, the cells were treated with or without 10 nM IGF-1 for another 24 h and fixed in 70 % ethanol for 12 h. Afterward, they were stained with propidium iodide, and the cell cycle distributions were determined using FACSCanto. Cell cycle distributions were further analyzed using FlowJo software. Each experiment was repeated at least three times. *P < 0.05; **P < 0.01; ***P < 0.0001

To further confirm how AGR2 induction contributes to IGF-1-induced breast cancer development, we investigated AGR2-knockdown effect on the IGF-1-induced cell proliferation, cell migration and cell cycle distribution (Fig. 5).

We performed an AGR2-knockdown experiment using specific short hairpin RNAs (shRNAs). MCF7 cells were transfected with viral vectors carrying AGR2 shRNA and were selected with puromycin for 14 days. Western blot assays confirmed that AGR2 protein levels were significantly reduced in two strains of MCF7 AGR2-knockdown cells (shAGR2-1 and shAGR2-2), compared with those in cells transfected with viral vectors alone (shControl) and normal MCF7 cells (Fig. 5a). Additionally, RT-PCR assays revealed the reduction of AGR2 at the mRNA level (Fig. 5b).

First, in an iCell proliferation assay, the data suggested that IGF-1 treatment remarkably enhanced shControl cell growth from 4 to 20 h. However, in shAGR2 cells, the curve’s slope did not show significant changes after IGF-1 exposure over the same period (Fig. 5c). Second, we performed a wound-healing assay on shControl and shAGR2-1/2 cells (Fig. 5d). Strikingly, the results showed that shControl cells migrated into the wound significantly faster than shAGR2 cells in the presence of IGF-1, whereas the migration rates in the absence of IGF-1 were almost equivalent. We further conducted a flow cytometry assay with each cell line with and without IGF-1 treatment for 24 h (Fig. 5e, f). The data revealed a significant reduction in the proportion of cell in G1 and an elevation of the proportion of cells in S phase for shControl cells under IGF-1 treatment, compared with those for shAGR2 cells. In all, we propose that the IGF-1-induced anti-estrogen drug resistance requires AGR2, because AGR2 is involved in the regulation of cell proliferation, migration and cell cycle progression by IGF-1.

## Discussion

AGR2, as a novel biomarker for breast cancer, has already become a recent research topic of interest. It has been widely reported that AGR2 is highly involved in cancer metastasis [18] and carcinogenesis [25]. However, in our study, AGR2 knockdown in MCF7 cells did not show a significant decrease in cell proliferation, migration or anti-estrogen drug resistance. AGR2 knockdown does play a significant role in IGF-1-induced cytological behaviors, which indicates that AGR2 function may strongly depend on the cancer microenvironment.

Our promoter analysis shown in Fig. 2 revealed that the region of M6, from −894 to −888 bp on the AGR2 promoter, whose specific element type and upstream pathway were not detected, is likely an ER-interdependent region in response to IGF-1. However, the regulatory intensity is less than the leucine zipper region and ERE. Therefore, we propose a transcriptional regulation model where the AGR2 promoter responding to IGF-1 stimulation focuses on the interaction between the three key elements, while the regulation mechanism of M6 deserves further investigation.

Our study also indicated that two cancer-related signal cascades—ERK1/2 and AKT—are located at the upstream of ER activation. We speculate that similar regulatory pattern may also apply to other growth factors such as EGF, VEGF, bFGF and PDGF in the regulation of AGR2 expression in ER-positive breast cancer, because these growth factors are widely reported to be related to ERK1/2, AKT or JNK pathway [26–28].

In Fig. 3a–f, AGR2 expression was effectively inhibited by ICI while ER phosphorylation was elevated by ICI. It is not a surprise because it has been reported that by binding and inhibiting ER, ICI itself could induce the phosphorylation of ER [29] (seen in Fig. 3b, e). Since AKT inhibitor and ERK1/2 inhibitor also abolish the IGF-1 signaling, for anti-ER drug-resistant tumor with an elevated AGR2 level, our study also indicated that targeting IGF-1/AGR2 pathway might be a treatment strategy.

The potential clinical significance of our study is the suggestion that evaluation of tumorigenesis risk in some conditions is related to IGF-1 overexpression. It has been reported that IGF-1 can be induced on certain circumstances, such as inflammatory bowel disease (IBD) [30], myocardial infarction [31], growth hormone stimulation [32] and diabetes [33]. Therefore, these diseases might generate a potential link to increased cancer risk due to the IGF-1 induction of AGR2.

Importantly, our results provide a novel mechanism of IGF-1-induced anti-estrogen drug resistance. We introduce AGR2 as a key modulator that is induced by IGF-1. The induction mechanism mainly lies in an ERE and in an AP-1 site on the AGR2 promoter, through the ERK, AKT and JNK pathway. Furthermore, the induction effect of IGF-1 to AGR2 leads to a promotion of cell proliferation and migration.

## References

[1] Thompson DA, Weigel RJ (1998). hAG-2, the Human homologue of the *Xenopus laevis* cement gland gene XAG-2, Is coexpressed with estrogen receptor in breast cancer cell lines. Biochem Biophys Res Commun.

[2] Kumar A, Godwin JW, Gates PB, Garza-Garcia AA, Brockes JP (2007). Molecular basis for the nerve dependence of limb regeneration in an adult vertebrate. Science.

[3] Van De Vijver MJ, He YD, van’t Veer LJ, Dai H, Hart AA, Voskuil DW (2002). A gene-expression signature as a predictor of survival in breast cancer. N Engl J Med.

[4] Wang Z, Hao Y, Lowe AW (2008). The adenocarcinoma-associated antigen, AGR2, promotes tumor growth, cell migration, and cellular transformation. Cancer Res.

[5] Wu Z, Wu Q, Ding X, Wang H, Shen Y, Fang S (2008). Expression of a novel metastasis-inducing protein human anterior gradient-2 (AGR2) in breast cancer and its clinical and prognostic significance. Zhonghua bing li xue za zhi Chin J Pathol.

[6] Takizawa Y, Taneike I, Nakagawa S, Oishi T, Nitahara Y, Iwakura N (2005). A panton–valentine leucocidin (PVL)-positive community-acquired methicillin-resistant *Staphylococcus aureus* (MRSA) strain, another such strain carrying a multiple-drug resistance plasmid, and other more-typical PVL-negative MRSA strains found in Japan. J Clin Microbiol.

[7] Liu D, Rudland PS, Sibson DR, Platt-Higgins A, Barraclough R (2005). Human homologue of cement gland protein, a novel metastasis inducer associated with breast carcinomas. Cancer Res.

[8] Bu H, Schweiger MR, Manke T, Wunderlich A, Timmermann B, Kerick M (2013). Anterior gradient 2 and 3—two prototype androgen-responsive genes transcriptionally upregulated by androgens and by oestrogens in prostate cancer cells. FEBS J.

[9] Zheng W, Rosenstiel P, Huse K, Sina C, Valentonyte R, Mah N (2005). Evaluation of AGR2 and AGR3 as candidate genes for inflammatory bowel disease. Genes Immun.

[10] Zhang Y, Ali TZ, Zhou H, D’Souza DR, Lu Y, Jaffe J (2010). ErbB3 binding protein 1 represses metastasis-promoting gene anterior gradient protein 2 in prostate cancer. Cancer Res.

[11] Zhang Y, Moerkens M, Ramaiahgari S, de Bont H, Price L, Meerman J (2011). Elevated insulin-like growth factor 1 receptor signaling induces antiestrogen resistance through the MAPK/ERK and PI3 K/Akt signaling routes. Breast Cancer Res.

[12] Myers MG, Sun XJ, White MF (1994). The IRS-1 signaling system. Trends Biochem Sci.

[13] He W, Craparo A, Zhu Y, O’Neill TJ, Wang L-M, Pierce JH (1996). Interaction of insulin receptor substrate-2 (IRS-2) with the insulin and insulin-like growth factor I receptors. Evidence for two distinct phosphotyrosine-dependent interaction domains within IRS-2. J Biol Chem.

[14] Tamura I, Kamada A, Goda S, Domae E, Yasunori S, Hayashi H (2006). Effect of insulin-like growth factor-I on the expression of EGR-1 and-2 in human periodontal ligament fibroblasts. J Oral Tissue Eng.

[15] Kahlert S, Nuedling S, van Eickels M, Vetter H, Meyer R, Grohé C (2000). Estrogen receptor α rapidly activates the IGF-1 receptor pathway. J Biol Chem.

[16] Campbell RA, Bhat-Nakshatri P, Patel NM, Constantinidou D, Ali S, Nakshatri H (2001). Phosphatidylinositol 3-kinase/AKT-mediated activation of estrogen receptor α. A new model for anti-estrogen resistance. J Biol Chem.

[17] Jiang B-H, Liu L-Z (2008). Role of mTOR in anticancer drug resistance: perspectives for improved drug treatment. Drug Resist Updates.

[18] Innes H, Liu D, Barraclough R, Davies M, O’neill P, Platt-Higgins A (2006). Significance of the metastasis-inducing protein AGR2 for outcome in hormonally treated breast cancer patients. Br J Cancer.

[19] Zweitzig DR, Smirnov DA, Connelly MC, Terstappen LW, O’Hara SM, Moran E (2007). Physiological stress induces the metastasis marker AGR2 in breast cancer cells. Mol Cell Biochem.

[20] Wright TM, Wardell SE, Jasper JS, Stice JP, Safi R, Nelson ER et al (2014) Delineation of a FOXA1/ERalpha/AGR2 regulatory loop that is dysregulated in endocrine therapy-resistant breast cancer. Mol Cancer Res. 2014:molcanres. 0195.2014. 10.1158/1541-7786.MCR-14-0195 PMC4272635 25100862

[21] Matys V, Kel-Margoulis OV, Fricke E, Liebich I, Land S, Barre-Dirrie A (2006). TRANSFAC^®^ and its module TRANSCompel^®^: transcriptional gene regulation in eukaryotes. Nucleic Acids Res.

[22] Stitt TN, Drujan D, Clarke BA, Panaro F, Timofeyva Y, Kline WO (2004). The IGF-1/PI3K/Akt pathway prevents expression of muscle atrophy-induced ubiquitin ligases by inhibiting FOXO transcription factors. Mol Cell.

[23] Ip YT, Davis RJ (1998). Signal transduction by the c-Jun N-terminal kinase (JNK)—from inflammation to development. Curr Opin Cell Biol.

[24] Honsho S, Nishikawa S, Amano K, Zen K, Adachi Y, Kishita E (2009). Pressure-mediated hypertrophy and mechanical stretch induces IL-1 release and subsequent IGF-1 generation to maintain compensative hypertrophy by affecting Akt and JNK pathways. Circ Res.

[25] Lepreux S, Bioulac-Sage P, Chevet E (2011). Differential expression of the anterior gradient protein-2 is a conserved feature during morphogenesis and carcinogenesis of the biliary tree. Liver Int.

[26] Arany I, Megyesi JK, Kaneto H, Price PM, Safirstein RL (2004). Cisplatin-induced cell death is EGFR/src/ERK signaling dependent in mouse proximal tubule cells. Am J Physiol Renal Physiol.

[27] Salameh A, Galvagni F, Bardelli M, Bussolino F, Oliviero S (2005). Direct recruitment of CRK and GRB2 to VEGFR-3 induces proliferation, migration, and survival of endothelial cells through the activation of ERK, AKT, and JNK pathways. Blood.

[28] Pratsinis H, Kletsas D (2007). PDGF, bFGF and IGF-I stimulate the proliferation of intervertebral disc cells in vitro via the activation of the ERK and Akt signaling pathways. Eur Spine J.

[29] Wakeling AE, Bowler J (1992). ICI 182,780, a new antioestrogen with clinical potential. J Steroid Biochem Mol Biol.

[30] Lawrance IC, Maxwell L, Doe W (2001). Inflammation location, but not type, determines the increase in TGF-β1 and IGF-1 expression and collagen deposition in IBD intestine. Inflamm Bowel Dis.

[31] Loennechen JP, Støylen A, Beisvag V, Wisløff U, Ellingsen Ø (2001). Regional expression of endothelin-1, ANP, IGF-1, and LV wall stress in the infarcted rat heart. Am J Physiol Heart Circ Physiol.

[32] Houseknecht K, Portocarrero C, Ji S, Lemenager R, Spurlock M (2000). Growth hormone regulates leptin gene expression in bovine adipose tissue: correlation with adipose IGF-1 expression. J Endocrinol.

[33] Kajstura J, Fiordaliso F, Andreoli AM, Li B, Chimenti S, Medow MS (2001). IGF-1 overexpression inhibits the development of diabetic cardiomyopathy and angiotensin II–mediated oxidative stress. Diabetes.

